# Supplementary material for: Changing Trends in Paralytic Shellfish Poisonings Reflect Increasing Sea Surface Temperatures and Practices of Indigenous and Recreational Harvesters in British Columbia, Canada
Source: Mar Drugs. 2021 Oct 14;19(10):568. doi: 10.3390/md19100568 (PMC8538720; doi:10.3390/md19100568)
Supplement: Supplementary file 1 [file marinedrugs-19-00568-s001.zip › marinedrugs-1374927-supplementary.pdf]

Report #	Date of Consumption	Incubation period range (hr)	No. ill (no. death)	Shellfish species consumed	Location description[a]	Harvest Area-Subarea[a]	Self-harvest or consumer Purchase (detail)	Shellfish sample (location) description: STX result as µg STX-eq 100 g^{-1} (date) (area was Closed or Open at time of harvesting)	Probable/ confirmed	Source of info
1	15 Jul 1793		3(1)	Mussels	Poison Cove, Mathieson Channel	7-7	Self-harvest (fishers)		Probable	[1]
2	2 May 1942	0.5	8(3)	Clams, mussels	Dodger Cove, Effingham Inlet, Ucluelet Harbour	23-7, 23-11	Self-harvest (FN)	Mussel (from area): 0.33cm^3 of shellfish flesh killed a mouse in 2-3 minutes<br>(Open)	Confirmed	[1]
3<br><br>PETS & WILDLIFE	23 Oct 1957		111(0)<br><br>CATS 1(7)<br>DOGS 2(0)<br>RACOONS (8)	Various, including oysters, mussels, clams	Comox area of Strait of Georgia	14-14	Self-harvest (locals)	Samples (from area): 19,840 mouse units equivalent to 3200 (Oct 24)<br>(Open)	61 Confirmed, 50 Probable	[1-4]
4	26 Jul 1964	2.5	7(0)	Butter clams	Evinrude Passage, Anger Island	5-16	Self-harvest (fishers)	Whole butter clams (from area): 1168, clams without necks: 880 (Aug 10).<br>(Closed)	Confirmed	[1]
5	31 May 1965		4(1)	Cockles	Theodosia Inlet, Malaspina Peninsula	15-4	Self-harvest (FN)	Leftover cooked cockles:1120 - 1920<br>Butter clams in area 3840 (Jun 1)<br>(Unknown)	Confirmed	[1, 4, 5]
6	May 1970		2(0)	Butter clams	Viner Sound, Gilford Island	12-38	Self-harvested (locals)	Butter clams (Health Bay, Gilford Island) 4200 µg/100g<br>(Unknown)	Confirmed	[4, 6]
7	20 Nov 1972	2-4	20(0)	Manila clams, oysters	Barkley Sound	23-8	Self-harvested (tourists)	Leftover cooked clams: 2900; raw clams: 2100-4000; raw oysters: 1300-1900<br>(Closed)	Confirmed	[7]
8	11 Jun 1975	0.3	1(0)[b]	Mussels	Work Channel, Prince Rupert	3-6	Self-harvested	Mussel (from area):12000<br>(Closed)	Confirmed	[7, 8]
9	Sept 1978	0.25	3(0)	Oysters	Fanny Bay, Vancouver Island	14-8	Self-harvested (tourists)	Leftover smoked oysters: 64<br>(Unknown)	Probable	[7]
10	16 May 1980	0.5	7(1)	Butter clams	Health Bay, Gilford Island	12-39	Self-harvested (FN)	Leftover raw butter clams: 8600; cooked butter clams: 3500; raw little neck clams: 2200<br>(Open)	Confirmed	[5, 7]
11	17 May 1980	12	3(0)	Butter clams	Shoal Harbour, Gilford Island	12-39	Self-harvested (FN)	Leftover raw butter clams: 2200<br>(Open)	Confirmed	[5]
12	Dec 1981		2(0)	Butter clams	Church House	13-18	Self-harvested (locals)	Raw clams (from area): 2400<br>(Open)	Confirmed	[7]
13	May 1982	0.25	5(0)	Mussels	Work Channel, Prince Rupert	3-6	Self-harvested (locals)	Leftover cooked mussels: 14000; raw: 26000-30000<br>(Closed, no signs posted)	Confirmed	[7]
14	8 May 1985	12	3(0)	Butter clams	Port McNeill, Simoom Sound	12-19	Self-harvested, (locals)	Biomonitored butter clams (from area): 9600 (May 7)<br>(Closed to butter clams, open to other species)	Confirmed	[6] PC
15	8 May 1985	Within hours	4(0)	Butter clams	Port McNeill, Simoom Sound, north of Echo Bay	12-19	Self-harvested, (tourists)	Biomonitored clams (from area): 9600 (May 7); stomach contents of case 42 µg; no STX detected in stomach contents of 3 cases.<br>(Closed to butter clams, open to other species)	Confirmed	[6] PC
16<br><br>PET	21 Apr 1987	2	1(0)<br><br>CATS 1(1)	Butter and littleneck clams.<br>Cats ate black tips of siphons	Porter's Beach near Wiltshire Road north of Chemainus, BC	17-6	Self-harvested (locals)	Leftover clams: 64-68; biomonitored butter clams (from area): 350 (March)<br>(Open)	Probable	[9, 10]

Report #	Date of Consumption	Incubation period range (hr)	No. ill (no. death)	Shellfish species consumed	Location description[a]	Harvest Area-Subarea[a]	Self-harvest or consumer Purchase (detail)	Shellfish sample (location) description: STX result as µg STX-eq 100 g^{-1} (date) (area was Closed or Open at time of harvesting)	Probable/ confirmed	Source of info
17	10 Oct 1988	5	1(0)	Oysters	Rendunda Sea Farms	Unknown	Purchased (restaurant)	N/A	Probable	PC
18	11 Oct 1988	6	3(0)	Clams	Kitkatla, north of Prince Rupert	5-3	Self-harvested (FN)	Biomonitored clams (from area): 300 (Nov 1) (Closed)	Probable	[11]; PC
19<br><br>PETS	25 Oct 1988	Between 0.25 -2.5	10(0)<br><br>CATS 1(2)	Butter clams	Edye Pass, Canoe Pass in Prince Rupert	4-4, 4-8	Self-harvested (FN)	Leftover clams (from Canoe Pass): 1300-1500; clams (from Edye Passage): 1700; one case 387 µg in gastric fluid and 131 µg in undigested solids (Closed)	Confirmed	[12, 13]
20	9 Apr 1989	10	1(0)	Rock scallops	Queen Charlotte City (hospital)	2-1	Self-harvested	N/A (Closed)	Probable	PC
21	17 Jun 1989	7	8(0)	Clams	Campbell River (hospital)	13-4	Self-harvest	N/A (Unknown)	Probable	PC
22	18 Aug 1989	Within 1 day	1(0)	Little neck clams	Unknown	Unknown	Purchased (retail)	Same batch clams (from store): not detected (Aug 21) (Unknown)	Probable	[14]
23	3 Dec 1989	0.25 - 2	6(0)	Butter clams	Flores Island near Hot Spring Cove	24-3	Self-harvested (FN)	Leftover butter clam chowder: 79 in broth; 230 in clam meat (Closed)	Confirmed	[15]
PET	27 Dec 1989		1(0)	Scallops	-	-	-	-		
24	20 Jun 1991	5	2(0)	Oysters and	Baynes Sound	14-8	Purchased (retail)	N/A (Open)	Probable	[16]
				Crab	Knights Inlet	12-29	Self-harvested (locals)	N/A (Open)		
25	20 Apr 1992	0.25	2(0)	Clams	Kingcome Inlet	12-43	Self-harvested (fishers)	Leftover raw cockles: 6700; butter clams 10,000 (Open, closed the following day)	Confirmed	[17]
26	7 Oct 1992	1	1(0)	Pink scallops (whole)	Egmont, Earle's cove	16-10	Self-harvested	N/A (Closed)	Probable	PC
27	30 Dec 1994		1(0)	Clams and mussels	Unknown	Unknown	Purchased (restaurant)	Same batch clams, mussels (from restaurant): <40 (Unknown)	Probable	[18]
28	5 Sep 1997	3	1(0)	East coast mussels	Nova Scotia	-	Purchased (retail)	Same batch mussels (4 sacks from store): <44 (Open)	Probable	[19]
PET	7 Aug 1997	4	1(0)	Mussels	Galiano Island, Saltspring	-	-	N/A	Probable	PC
29	11 Oct 1997	2	3(0)	Pink and spiny scallops	Canoe Rock, Portland Island (park)	18-6	Self-harvested (locals)	N/A (Closed to all bivalve shellfish due to toxic PSP bloom on Sep 5. No signs posted.)	Confirmed	[20]
30	16 Oct 1997		2(0)	Oysters, butter clams	Kulleet Bay, Ladysmith	17-5	Self-harvested (FN)	Biomonitored mussels (Coffin Point): >5000 (Oct 13) (Closed, harvest area under prohibition due to toxic bloom.)	Confirmed	[21, 22]
31	2 Jan 1998	1.5 – 2	8(0)	Butter clams	Coffin Point near Ladysmith	17-5, 17-6, 17-7	Self-harvested (FN)	Biomonitored butter clams (3 different locations in Area 17): 580-810 (early Dec) (Closed, harvest area under prohibition for all bivalve species except littleneck clams, manila clams, and oysters due to toxic bloom)	Confirmed	[23]
32	May 1999		2(0)	Scallops	Herbert Inlet	24-4	Self-harvested (employees)	N/A (Closed May 20)	Probable	[24]; FN0315
33	8 Dec 2002	0.5	1(0)	Butter clams	Rivers Inlet, Port McNeill	9-6	Self-harvested	N/A, no testing in area 9 in 2002. (Closed)	Probable	PC; FN1165
34	31 Jul 2003		6(0)	Mussels	Effingham Inlet	23-6	Purchased (retail)	Leftover mussels: 2100 (Closed)	Confirmed	[25-27]; FN0517

Report #	Date of Consumption	Incubation period range (hr)	No. ill (no. death)	Shellfish species consumed	Location description[a]	Harvest Area-Subarea[a]	Self-harvest or consumer Purchase (detail)	Shellfish sample (location) description: STX result as µg STX-eq 100 g^{-1} (date) (area was Closed or Open at time of harvesting)	Probable/ confirmed	Source of info
35	23 May 2004		9(0)	Clams, cockles	Porpoise Bay, Sechelt (park)	16-5	Self-harvested (campers)	N/A in 16-5. Biomonitored mussels (adjacent harvest area 16-6 Storm Bay): 200 (May 21), 1600 (May 16), 3500 (Jun 2) (Closed; no signs posted)	Probable	PC; [28] FN0290
36	5 Jun 2004	6	1(0)	Mussels	Unknown	Unknown	Purchased (restaurant)	N/A (Unknown)	Probable	PC
37	5 Apr 2005		6+(0)	Butter clams	Cowichan	18-7, 18-8	Self-harvested (FN)	Biomonitored mussels (from area): <44 (Mar 14, Apr 12) (Closed to butter clams)	Probable	[29-31]
38	18 Sep 2006	4	1(0)	Crab	Tofino	24-9	Self-harvested (fisher)	Biomonitored mussels (from area): <42 (Sep 18); (adjacent harvest area 24-8): 44 (Sep 18). (Open to all bivalves except butter clams and scallops)	Probable	[32]; FN0868
39	25 Sep 2008	1	2(0)	Clams (canned)	Product of Thailand	-	Purchased (retail)	Leftover samples: <40 µg/100g	Probable	[33, 34]
40	6 Nov 2009	0.25	1(0)	Mussels	Tonovin Beach, Tofino (park)	24-8	Self-harvested (local campers)	Biomonitored mussels (from area): 130 (Sep 27); 67 (Oct 4); <44 (Oct 12, 19, Nov 1, 15) (Closed)	Confirmed	[35] FN0907
41	13 Jun 2010		4(0)	Cockles	Bamberton Beach (park)	19-8	Self-harvested (campers)	Leftover cockles and raw littleneck clams (from area): ,44; biomonitored mussels (from area): <44 (Jun 9, 14); 97 (Jun 22); 410 (Jun 28) (Open to some species: littleneck, manila clams, oysters, mussels. Close to other bivalve species)	Probable	Database[A]; FN0418
42	29 Sep 2011	3 – 96	2(0)	Mussels	Okeover Inlet	15-4	Purchased (retail)	Biomonitored mussels (from area): < 40 (Sep 25), 40 (Oct 1); 330 (Oct 8) (Open to all shellfish species except butter clams)	Probable	[36] Database[A]; FN0926
43	12 Oct 2011		1(0)	Mussels	Okeover Inlet	15-4	Purchased (restaurant)	Biomonitored mussels (from area): 40 (Oct 1); 330 (Oct 8); 790 (Oct 14) (Open to all shellfish species except butter clams. Closed to all bivalves on Oct 14)	Confirmed	[36] Database[A]; FN1005; FN1028
44	17 Jul 2012		4(0)	Manila clams, oysters	Effingham and Useless Inlets, Sechart Channel	23-6, 23-8	Self-harvested (locals, tourists)	Biomonitored mussels (from area 23-6): <25 (Jul 10), 120 (Jul 16), 5200 (Jul 17), 62 and 3000 (Jul 19); (from area 23-8): 28 (Jul 8), 34 (Jul 17), <25 (Jul 19) (Open to some species (Jul 5): manila, littleneck clams, oysters, mussels. Closed to other bivalve species. Closed to all species (Jul 18)	Confirmed	[37]; Database[A, B]; FN0553, FN0610
45	5 Dec 2013	3	1(0)	Clams	Silva Bay, Gabriola Island (park)	17-10	Self-harvested (boaters)	N/A. Self-harvested in Aug, frozen. Biomonitored geoduck: <25 (Apr 21, last sample report for 2013) (Open to some species: littleneck, manila, geoduck, horse clams, oysters, mussels. Closed to other bivalve species)	Probable	PC; Database[A]; FN1180
46	18 Jan 2014	1	1(0)	Clams	Gabriola Island (park)	17-10	Self-harvested (camper)	N/A (no biomonitored samples reported for 2014) (Open to some species: manila, littleneck, geoduck, horse clams, oysters mussels. Closed to other bivalve species)	Probable	PC; FN0040
47	18 Mar 2015		1(0)	Clams	Haida Gwaii area	2-1	Self-harvested	Biomonitored mussels: <25 (Jun 5, first test of harvest area that year) (Closed)	Probable	PC; FN0424
48	16 May 2015	0.25	1(0)	Mussels	Mystic Beach, Juan de Fuca (park)	20-4	Self-harvested (campers)	Beach mussels (from area): <25 (May 26); biomonitored mussels <25 (May 12, May 20) (Open to some species: littleneck, manila clams, oysters, mussels. Closed to other bivalve species)	Probable	PC; Database[A]; FN0424

Report #	Date of Consumption	Incubation period range (hr)	No. ill (no. death)	Shellfish species consumed	Location description[a]	Harvest Area-Subarea[a]	Self-harvest or consumer Purchase (detail)	Shellfish sample (location) description: STX result as µg STX-eq 100 g^{-1} (date) (area was Closed or Open at time of harvesting)	Probable/ confirmed	Source of info
49	23 May 2015	0.5	2(0)	Mussels and clams	2 sources implicated BC and Prince Edward Island (PEI)	14-8 and PEI	Purchased (retail)	Biomonitored mussels (from area) <25 (May 18, 25) (Open. Area 14-8 open to all bivalves except butter clams)	Probable	PC; Database[A]; FN0451
50	2 Jul 2015	1.5	1(0)	Oysters	West Roscoe Bay	15-5	Self-harvested (recreational, boater)	Biomonitored mussels (from area): <25 (Jun 28, Jul 6). (Open to some species: manila, littleneck clams, oysters, mussels, scallops. Closed to other bivalve species)	Probable	PC; Database[A]; FN0610
51	8 May 2016	1	1(0)	Mussels	Unknown	Unknown	Purchased (restaurant)	N/A (Unknown)	Probable	PC
52	10 Jul 2016	0.5	2(0)	Clams	Read Island	13-17	Purchased (farmers' market)	Biomonitored mussels (from area): 120 (Apr 27), 110 (May 5), <25 (Jun 29, Jul 7); biomonitored oysters (from area): <25 (Jun 6) (Open to some species: manila, littleneck, varnish clams, oysters, mussels. Closed to other bivalve species)	Probable	PC; FN0635
53	5 Sep 2016	6.0	4(0)	Mussels	Newfoundland	-	Purchased (restaurant)	Same batch (from restaurant): <25 (Open)	Probable	Database[B,C]
54	14 Jan 2017	4	1(0)	Clams	BC (Unknown)	Unknown	Purchased (retail)	N/A (Unknown)	Probable	PC; Database[C]
55	24 May 2017	1	1(0)	Clams	Rathtrevor Beach, Parksville (park)	14-1	Self-harvested (local)	Biomonitored mussels (from area): <25 (Jun 12, first test of harvest area that year) (Closed)	Probable	PC; FN0450
56	22 Jun 2017		1(0)	Crab	Unknown	Unknown	Self-harvested	N/A (Unknown)	Probable	PC
57	20 Aug 2017	12	1(0)	Clams	Porpoise Bay, Sechelt (park)	16-5	Self-harvested	Biomonitored geoduck clams (from area): <25 (Jul 21, last date of sampling from this area in 2017); biomonitored mussels (adjacent area 16-6): <25 (Aug 13, Aug 25) (Open to some species: manila, littleneck clams. Closed to other bivalve species)	Probable	PC; FN0830
58	11 Mar 2018	3	1(0)	Oysters	Okeover Inlet	15-4	Purchased (retail store)	Biomonitored mussels (from area): <25 (Feb 26, Mar 12) (Open to some species: manila, littleneck clams, oysters, scallops. Closed to other bivalve species)	Probable	PC; FN0176
59	20 Jun 2018	2.0	4(0)	Mussels	Saanich Inlet	19-8	Self-harvested (FN)	Leftover mussels (from soup): 830 (Closed)	Confirmed	PC; Database[B,C]; FN0499
60	15 Sep 2018	1.5	2(0)	Mussels	Marina Island and a WA state source	13-15 and WA state	Purchased (restaurant)	Biomonitored mussels (from area): <25 (Sep 9, 16); STX not detected in US source. (Open to some species: manila, littleneck, geoduck and horse clams, oysters, mussels. Closed to other bivalve species)	Probable	PC; FN1000
61	29 Oct 2018	0.25	1(0)	Mussels	Sechelt inlet	16-6	Self-harvested (fisher)	Leftover mussels (from soup): 20,000; frozen mussels: 15,000 (Closed)	Confirmed	PC; Database[C]; FN197
62	13 Mar 2019	1.5	3(0)	Butter clams	Dundas Island	4-1	Self-harvested (FN)	Leftover butter clams (from 2018 harvest, frozen): 1300 (siphon), 180 (gut) of clams. Urine results: >116 ng STX eq./mL urine and 25 ng STX eq/mL urine (Closed)	Confirmed	Northern HA notification; Database[B,C]

Report #	Date of Consumption	Incubation period range (hr)	No. ill (no. death)	Shellfish species consumed	Location description[a]	Harvest Area-Subarea[a]	Self-harvest or consumer Purchase (detail)	Shellfish sample (location) description: STX result as µg STX-eq 100 g^{-1} (date) (area was Closed or Open at time of harvesting)	Probable/ confirmed	Source of info
63	4 Aug 2020	0.5	1(0)	Oysters	Saltspring Island	17-9 or 18-7	Self-harvested (local)	Biomonitored mussels (from 17-9): 78 (Jul 29), <25 (Aug 10); biomonitored mussels (from 18-7): 180 (Jul 30), 85 (Aug 5), 43 (Aug 12) (Closed)	Confirmed	PC; FN0742

[a] – Location descriptions and harvest sub-areas may be visualized on the BCCDC Biotoxin and Sanitary Contamination Closures Map for Shellfish Harvesting in British Columbia at maps.bccdc.ca/shellfish
Databases: A=BCCDC shellfish related illness database (1994-2018); B= Canadian Network for Public Health Intelligence database; C=Panorama database (2016 to present);
PC – BC Drug and **P**oison Information **C**entre call records; FN – Fishery Notices from Fisheries and Oceans Canada, https://notices.dfo-mpo.gc.ca/fns-sap/index-eng.cfm?pg=fishery_search&ID=all to assess area closures [b] – Citations differ, [8] reports a single illness, [7] reports two illnesses.

REFERENCES

1. Quayle, D.B., *Paralytic shellfish poisoning in British Columbia*, in *Bulletin 168*. 1969, Fisheries Research Board of Canada: Nanaimo, BC. p. 68 p.
2. Taylor, F.J.R. and P.J. Harrison, *Harmful algal blooms in western Canadian waters*, in *PICES Scientific Report*, F.J.R. Taylor and V.L. Trainer, Editors. 2002. p. 77-88.
3. Anderson, L., *Toxic shellfish in Briitsh Columbia.* American Journal of Public Health and the Nations Health, 1960. **50**(1): p. 71-83.
4. BC Ministry of Environment, *Red tide - what is it all about?*, S.M.a.D. Section, Editor. p. 1-5.
5. Heimann, A., et al., *Paralytic shellfish poisoning in British Columbia - Summer 1980*, in *Disease Surveillance*, P.S. Epidemiology, Editor. 1980, BC Ministry of Health. p. 1-6.
6. Bell, P., R. Hicks, and J. Millar, *Paralytic shellfish poisoning - Port McNeill area, Vancouver Island, British Columbia.* Canada Diseases Weekly Report, 1985. **11-35**: p. 149-50.
7. Todd, E., *Shellfish and fish poisoning in Canada, 1972-1983.* Canada Diseases Weekly Report, 1984. **10-6**: p. 21-24.
8. Jackson, K., et al., *Paralytic Shellfish Poisoning - British Columbia.* Canada Diseases Weekly Report, 1976. **2-4**: p. 16.
9. Rideout, A., *Paralytic shellfish poisoning - central Vancouver Island health unit*, in *Disease Surveillance*. 1988. p. 176.
10. Rideout, A. and E. Todd, *Paralytic shellfish poisoning - British Columbia.* Canada Diseases Weekly Report, 1988. **14-13**: p. 53-55.
11. Fisk, B., *FAX Re: PSP Poisonings (Forwarded note from D. Bowering) TO:*, A. Hazelwood, Editor. 1988: Skeena #16, Terrace, BC.
12. Stroh, D., D. Bowering, and C.R. Armstrong, *Paraltyic shellfish poisoning (PSP)*, in *Disease Surveillance*. 1988, Epidemiology Prevention Services: Victoria, BC. p. 299-301.
13. *Butter clams confirmed as cause of illness*, in *The Vancouver Sun*. 1988, Postmedia Network Inc.: Canada, Vancouver, B.C. p. A14.
14. Copley, B.D. and A. Hazelwood, *A case of paralytic shellfish poisoning*, in *Community and Family Health*. 1989. p. 1.
15. Nickel, V., *Report on paralytic shellfish poisoning - butter clams from Clayoquot Sound region*. 1989, Central Vancouver Island health unit. p. p. 3.
16. Preston, T., *Oyster complaint - suspected PSP (Letter to J. Pinn, Fisheries & Oceans)*, P.H. Protection, Editor. 1991, Ministry of Health. p. p. 1.
17. Todd, E., et al., *An outbreak of sever paralytic shellfish poisoning in British Columbia.* Canada communicable disease report = Releve des maladies transmissibles au Canada, 1993. **19**(13): p. 99-102.
18. Werker, D.H., *Update - paralytic shellfish poisoning (FAX) to All health units & Chief Environmental Health Officers*. 1994.
19. Oikawa, G., *Briefing note consumer complaint #62-970908-01 possible PSP poisoning (to T. Preston)*. 1997, Canadian Food Inspection Agency: Burnaby, BC.
20. *Outbreak of shellfish poisoning*, in *Gulf Islands' Island Tides*. 1997.
21. Cleverley, B., *Red tide prompts warning*, in *Times Colonist*. 1998: Victoria, BC. p. A6.
22. Schallie, K., *PSP illness report Oct 22, 1997 (to S. Liem)*. 1997, Canadian Food Inspection Agency: Burnaby, BC. p. 1 p.
23. Schallie, K., *PSP illness report Jan 7, 1998 (to S. Liem)*. 1998, Canadian Food Inspection Agency: Burnaby, BC. p. 1 p.
24. Nations, F.a.A.O.o.t.U. *Marine biotoxins, Cases and outbreaks of PSP 2.7.4 North America* 2004 [cited 2021 Sep 28]; Available from: http://www.fao.org/3/y5486e/y5486e0c.htm#bm12.

25. Canadian Food Inspection Agency, *20030803-Health hazard alert (paralytic shellfish toxins)/Avertissement de danger pour lat sante (phycotoxines paralysante)*. 2003: Ottawa, ON. p. 1.
26. Walton, P., *Fish store fined $2,000: Sea Drift Market sold red tide clams*, in *Nanaimo Daily News*. 2006, Postmedia Network Inc.: Canada, Nanaimo, B.C. p. A3.
27. Wilson, V., *Shellfish harvester under investigation*, in *Nanaimo Daily News*. 2003, Postmedia Network Inc.: Canada, Nanaimo, B.C. p. A.3.
28. Luba, F., *Nine seek treatment after eating shellfish*, in *The Province*. 2004, Postmedia Network Inc.: Canada, Vancouver, B.C. p. A4.
29. Watts, R., *Two jailed for selling tainted fish*, in *Times-Colonist*. 2007, Postmedia Network Inc.: Canada, Victoria, B.C. p. B5.
30. *Duncan man to face charges of selling illegally caught fish that left two people ill*, in *The Vancouver Sun*. 2005: Canada, Vancouver, B.C.
31. *Charges added in shellfish poisoning*, in *Cowichan News Leader*. 2005, Torstar Syndication Services a Division of Toronto Star Newspapers Limited: Canada, Duncan, B.C. p. 8.
32. Rodriguez-Maynex, L., *PSP report - BC waters (to E. Galanis)*. 2006, Canadian Food Inspection Agency: Burnaby, BC.
33. Mosely, G., *Suspected foodborne intoxication complaint/report*, L. Copeland, Editor. 2008, Interior Health Authority: Cranbrook, BC. p. 3.
34. Hornby, L., *Results for clam product sampled during illness investigation*, G. Mosely, Editor. 2008, Government of Canada: Burnaby, BC. p. 1.
35. Cordner, K., *PSP*, M. Taylor, Editor. 2009, Vancouver Island Health Authority: Courtenay, BC. p. 2.
36. Canadian Food Inspection Agency, *20111015 - HEALTH HAZARD ALERT (Paralytic Shellfish Poisoning) / DANGER POUR LA SANTE (Intoxication par phycotoxine paralysant)*. 2011, Government of Canada: Ottawa, BC.
37. McIntyre, L. and T. Kosatsky, *Shellfish poisonings in BC: commerical product as source.* BCMJ, 2013. **55**(6): p. 290-91.
